# Type I Sialidosis in a Chinese family: a case report and literature review

**DOI:** 10.1186/s42494-025-00225-3

**Published:** 2025-06-04

**Authors:** Xia Zhou, Shengyou Su, Shenghua Li, ZuFang Yi, Liling Feng, Junyi Chen, Binglin Fan

**Affiliations:** 1 Department of Neurology, the People’s Hospital of Guangxi Zhuang Autonomous Region, Nanning, 530021 China; 2 Department of Neurology, the First People’s Hospital of Qinzhou, Qinzhou, 535099 China; 3 Department of Geriatric Neurology, the People’s Hospital of Guangxi Zhuang Autonomous region, 530021, Nanning, China

**Keywords:** Sialidosis, *NEU1* gene, Seizure, Ataxia, Exome sequencing

## Abstract

**Background:**

Sialidosis is an autosomal recessive hereditary disease characterized by the mutation of neuraminidase-1 (*NEU1*) gene, resulting in decreased activity of α-N-acetylneuraminidase. This leads to metabolic abnormalities in various organs. Sialidosis is classified into two distinct clinical phenotypes, type I and type II, based on the age of onset and severity of clinical manifestations.

**Case presentation:**

Here, we report a case involving a patient and his two sisters, all of whom showed seizures and ataxia during adolescence, with progressively worsening symptoms. Prior to admission, none of the patients had received a systemic diagnosis or treatment. The whole exome sequencing identified a homozygous *NEU1* mutation (NM_000434.3:c.544A > G [p.Ser182Gly]) in all three siblings. Their parents and children, who were asymptomatic, were found to be heterozygous carriers. The three patients were ultimately diagnosed with type I sialidosis and treated with antiseizure medications, but they continued to experience recurrent seizures.

**Conclusions:**

This case report enhances our understanding of sialidosis, particularly in patients presenting with seizures and ataxia. Furthermore, the gene sequencing is a crucial tool for confirming the diagnosis of sialidosis and provides a valuable approach for genetic counseling in affected families.

## Background

Sialidosis is an autosomal recessive genetic disorder caused by mutations in the *NEU1* gene, which is located on chromosome 6p21.33 [1]. The *NEU1* gene encodes neuraminidase-1, an enzyme that is localized in the lysosomes and plasma membranes of various tissue cells, where it plays a key role in the degradation of N-acetylneuraminic acid (sialic acid), a major component attached to the non-reducing end of sugar chains in glycoproteins and glycolipids [2]. Mutations in *NEU1* lead to a reduction in α-N-acetylneuraminidase activity, causing abnormal accumulation of sialic acid-containing oligosaccharides, glycoproteins, and glycolipids in tissues, such as liver, brain, kidney, bone marrow, thyroid, and adrenal glands [3]. These abnormal accumulation affect the function of organ and tissue.

The incidence of sialidosis is rare, with an estimated prevalence of approximately 0.04 per 100,000 individuals [4]. However, due to the complex clinical manifestations, the incidence may have been underestimated. Sialidosis is clinically classified into two types based on the age of onset and the severity of symptoms [5]. Type I sialidosis usually manifests clinical symptoms during adolescence or adulthood, with the median diagnostic age of 21 years. These patients generally exhibit normal physical development and intelligence. And common clinical signs include impaired vision, myoclonus, ataxia, hyperreflexia and seizures. A distinctive feature of type I sialidosis is the presence of a cherry-red spot in the fundus of the eye. In contrast, type II sialidosis is associated with complete loss of α-N-acetylneuraminidase activity [6]. Patients with type II sialidosis experience similar neurological symptoms to those seen in type I patients, but the condition is more debilitating, with additional features such as severe psychomotor retardation, skeletal dysplasia, facial roughness, hepatosplenomegaly, hydrops fetalis, mental retardation, and juvenile mortality [7]. Sialidosis is progressive, leading to significant deterioration in the quality of life, including dyskinesia and reduced life expectancy [8].

This report presents a case involving three family members with type I sialidosis who exhibited similar clinical manifestations (seizures, myoclonus and ataxia) and were found to carry a homozygous *NEU1* mutation (NM_000434.3:c.544 A > G[p.Ser182Gly]). The diagnosis of type I sialidosis in these patients underscores the importance of genetic testing for early detection, which can aid in genetic counseling and potentially reduce the incidence of the disease within this family.

## Case presentation

A 33-year-old male patient was admitted to the hospital on May 23, 2023. At the age of 16, he experienced only one episode of generalized tonic–clonic seizures throughout the course of the disease. Over the following two years, he developed an unsteady gait, which did not initially impact his physical activities, along with verbal fluency difficulties. At the same time, he experienced myoclonic jerks in both his upper and lower limbs approximately 10 episodes per day. These symptoms progressively worsened. By the age of 20, the frequency of myoclonic jerks had increased to 20–30 episodes per day, and the patient had difficulty in walking and frequently falling. However, he did not receive any systemic treatment at this stage. At 29 years of age, the patient began antiseizure therapy with valproate (100 mg three times daily), but the frequency of myoclonic jerks did not decrease. Two years ago, the patient visited a local hospital, where an electroencephalogram (EEG) revealed extensive spike and wave discharges, and a magnetic resonance imaging (MRI) scan of the head showed no abnormalities. The patient was prescribed valproate (200 mg three times daily) and levetiracetam (500 mg twice daily) by the local hospital, but his symptoms persisted without significant improvement.

 Upon admission to our hospital, the patient exhibited myoclonic seizure (10–20 times per day), obvious unsteady gait, diplopia and speech difficulties. Physical examination revealed normal memory, calculation and orientation abilities. He presented with fluency disorder, myoclonic jerks, clumsiness in finger-nose and heel-knee-shin tests, Romberg test (+), straight-line walking test (+). No abnormalities were observed in his cranial nerves, muscle strength, Babinski sign, or limb tendon reflexes.

**Fig. 1:**
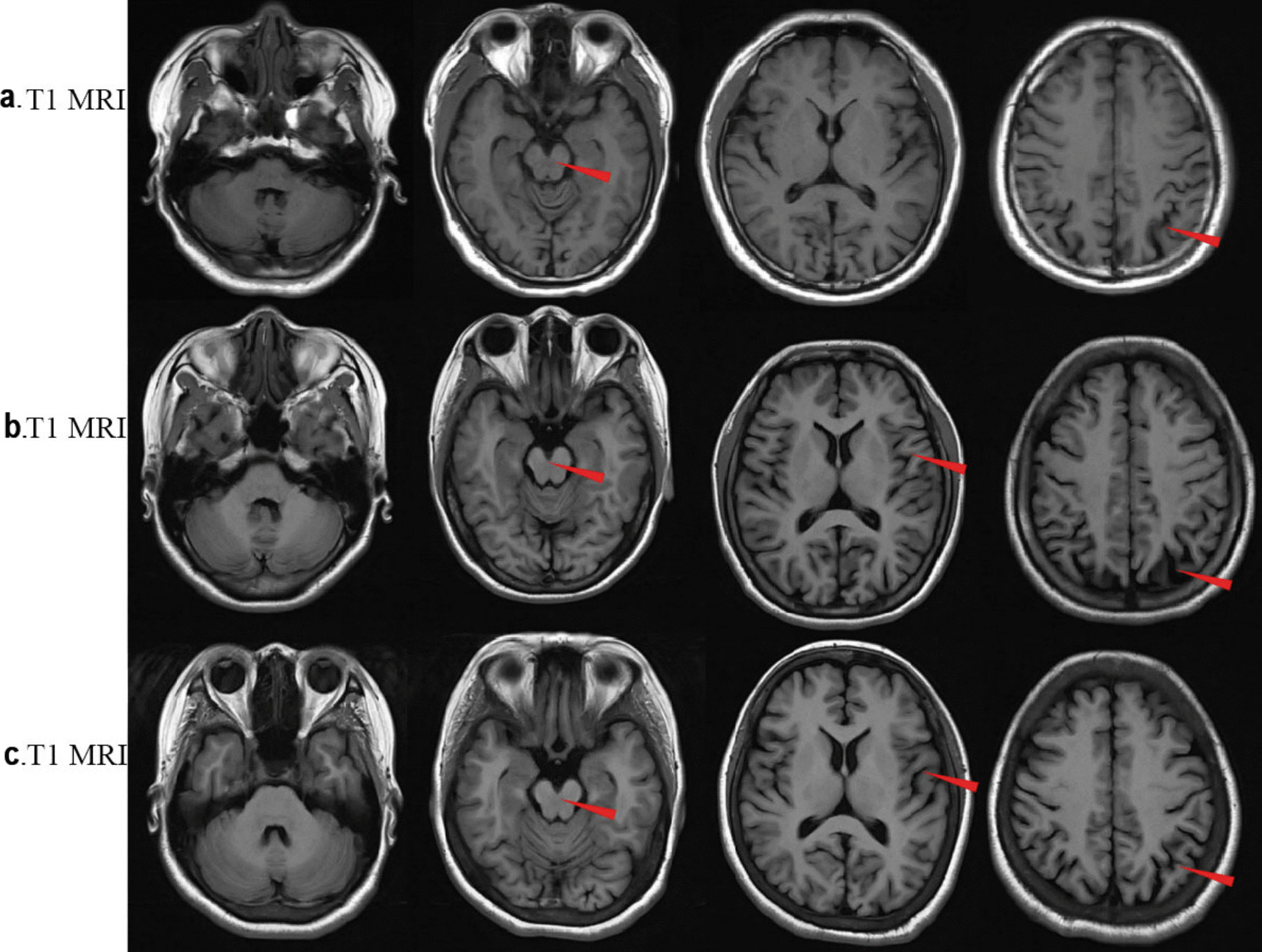
Axial T1 brain magnetic resonance imaging (MRI) of the patients both show brain atrophy. a. the reported patient. b. The second elder sister of the patient; c.The eldest sister of the patient

**Fig. 2:**
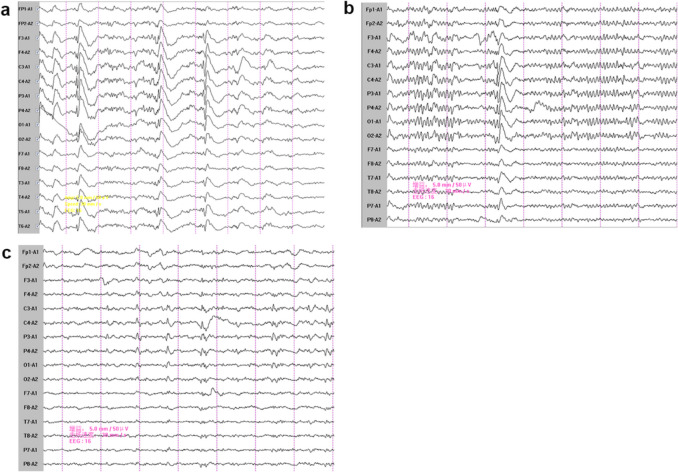
Electrocephalogram (EEG) recording of the patients show sharp waves or spike slow waves in the patients. a. The reported patient. b. The second elder sister of the patient; c. The eldest sister of the patient

There was no significant abnormalities in the laboratory tests, including electrolytes, thyroid function, myocardial enzyme, liver function, kidney function, hepatitis B surface antigen, antibodies to treponema pallidum and HIV. B-ultrasound of thyroid, heart and abdomen also revealed no significant findings. MRI of the head showed cerebral atrophy with no abnormal signal or heterotopic structure (Fig. 1a). Electroencephalogram showed spike-slow complex waves in bilateral hemispheres (Fig. 2a). Cerebralspinal fluid (CSF) analysis showed increased CSF pressure (200 mmH_2_O, reference range 80–180 mmH_2_O), a normal protein level (344 mg/L, reference range 150–450 mg/L), leucocytosis (42 × 10^6^/L, reference range < 8 × 10^6^/L), erythrocytosis (30 × 10^6^/L, reference range < 5 × 10^6^/L), normal chloride (127 mmol/L, reference range form 120–132 mmol/L) and glucose (4.37 mmol/L, reference range 2.8–4.5 mmol/L), with no evidence of pathogen infection. Funduscopic examination was normal, without macular cherry-red spots. Negative imaging revealed paralysis of the right superior rectus muscle.

**Fig. 3:**
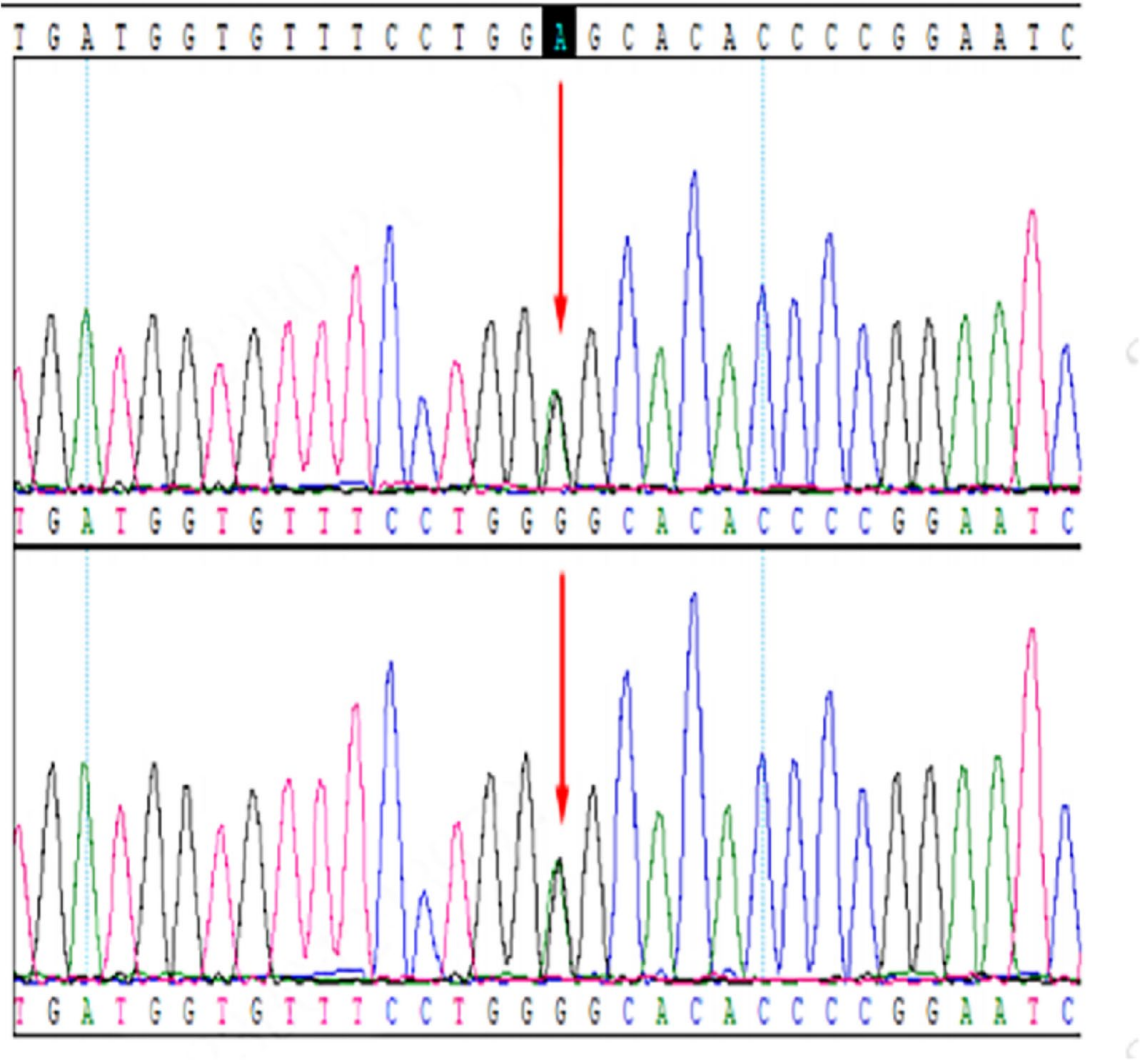
The gene sequencing results indicated a heterozygous mutation in *NEU1* in the patients’ father and mother (*NEU1*; NM_000434.3;c.544 A > G;p.Ser182Gly)

**Fig. 4:**
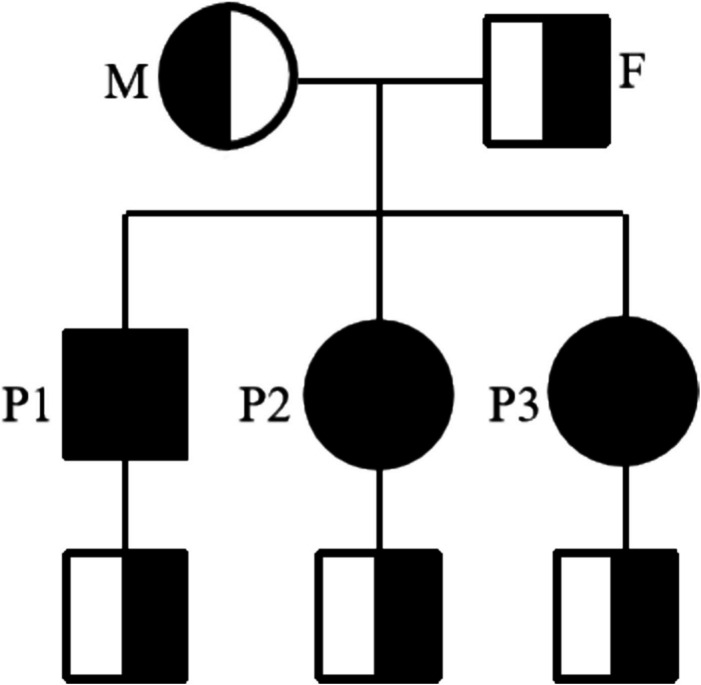
Family pedigree showing the affected individuals. F, father; M, mother; P1, the reported patient; P2, the second elder sister of the patient; P3, the eldest sister of the patient

The patient was born in a non-consanguineous family and had a normal growth and developmental history, having completed high school. Both of his two sisters exhibited similar symptoms, including generalized tonic–clonic seizures, myoclonic jerks, unsteady gait and speech difficulties, with more severe manifestations than the patient. Their EEGs showed epileptiform discharges (Fig. 2b and 2c), and their brain MRIs revealed more severe cerebral atrophy than the patient (Fig. 1b and 1c). Both the patients’ parents and their respective children were asymptomatic. Informed consent for genetic testing was obtained from the family. Whole-exome sequencing revealed a homozygous mutation in the *NEU1* gene (NM_000434.3:c.544 A > G, p.Ser182Gly) in the patient and his two sisters (Fig. 3). Three boys, the respective offspring of the patient and his two sisters, as well as the patient’s parents, were found to carry a heterozygous *NEU1* mutation (Fig. 4). Based on the clinical symptoms and genetic results, the patient and his two sisters were diagnosed with type I sialidosis.

The patient was started on a treatment regimen consisting of valproate (400 mg three times daily), levetiracetam (750 mg twice daily), and perampanel hydrate (2 mg daily). After the above treatment, the frequency of his myoclonic attacks was reduced.

## Discussion

Type I sialidosis is an autosomal recessive inherited disorder with diverse clinical manifestations, often lacking distinctive features that can easily lead to misdiagnosis or missed diagnosis. In the reported family, despite the clear presence of seizures and ataxia in the patients, the final diagnosis of type I sialidosis was delayed until the *NEU1* gene mutation (NM_000434.3:c.544 A > G[p.Ser182Gly]) was detected. This genetic case facilitates a better understanding of type I sialidosis, and strengthen the awareness of genetic detection.

Patients with type I sialidosis often present with a variety of neurological symptoms indicating central nervous system damage. Sialic-acid-containing proteins have been found enriched in neurons within the cerebral cortex, thalamus and cerebellum in type I sialidosis [9, 10]. The abnormal deposition of sialic-acid proteins in the central nervous system leads to both functional and structural brain damage. MRI scans of the three patients in this study revealed significant cerebral atrophy, including the temporal lobe, frontal lobe, cerebellum, and brainstem, consistent with previous reports. A Japanese patient with sialidosis type I showed rapid diffuse brain atrophy with increasing age, particularly in the temporal lobe, cerebellum and brainstem [11]. This MRI pattern was similar to a female patient who showed mild enlargement of the fourth ventricle at age 21, which progressed to severe global brain atrophy by age 40 [12]. However, in some patients, brain MRI may remain normal or show only mild changes even after 10 years of disease progression. In addition to brain damage, patients with type I sialidosis may also present with distinctive cherry-red spots in the eyes. Notably, this patient presented with diplopia and paralysis of the right superior rectus muscle. This indicates not only central nervous system but also peripheral neuropathy are injured. However, the funduscopic examination was normal. This underscores the importance of long-term follow-up for the patient to monitor potential progression and the development of new symptoms over time.

Type I sialidosis is a rare autosomal recessive genetic disorder caused by mutation in the *NEU1* gene. With advancements in gene sequencing technology, it has become increasingly accessible to clinicians and patients, facilitating timely and accurate diagnosis of genetic diseases [13, 14]. Due to the late-onset, mild, or atypical symptoms of type I sialidosis, it often leads to misdiagnosis or missed diagnosis. Therefore, genetic testing is crucial to identify pathogenic *NEU1* mutations. Moreover, The *NEU1* mutation also exhibits genetic heterogeneity across different populations, with the c.544 A > G mutation being the most common. Studies by Lv et al. have shown that this mutation is more prevalent in China compared to other regions, such as Asia, Europe and North America [15]. In mainland China, the c.544 A > G mutation accounts for approximately 75% of cases of type I sialidosis. In the present study, all patients carried the same c.544 A > G mutation, yet displayed different levels of disease severity, which may be linked to the extent of enzyme deficiency and subsequent enzyme editing abnormalities [6]. Additionally, the mutation was detected not only in the affected individuals but also in their heterozygous parents and the next generation, suggesting a familial inheritance pattern. Therefore, genetic testing for *NEU1* mutations is essential for early diagnosis, intervention, genetic counseling, prognostic prediction, and the potential for future gene therapies.

The conventional treatment of type I sialidosis is palliative care based on clinical symptoms. One of the features of type I sialidosis is refractory seizures, which are often difficult to control despite systematic antiepileptic therapy. As reported by Odaka et al., neurons in patients with sialidosis exhibit augmented α-amino-3-hydroxy-5-methyl-4-isoxazole-propionate receptor (AMPAR)-mediated Ca^2+^ influx, which contributed to seizure activity [16]. A novel antiseizure medication, perampanel, has been shown to effectively suppress the AMPAR-mediated excess Ca^2+^ influx, and it has been demonstrated to significantly reduce myoclonic convulsion attacks in patients with type I sialidosis. In this study, patients diagnosed with type I sialidosis were treated with perampanel, leading to a notable decrease in seizure frequency. Thus, perampanel should be given further attention as a promising treatment option for managing seizures in type I sialidosis. Due to the limited treatments, new practical methods are also constantly being researched in type I sialidosis. Mosca et al. found that the romidepsin and betaine can increase *NEU1* activity in mice and cell models [17]. Moreover, animal models for both type I and type II sialidosis have been developed, providing an essential platform for investigating new treatment strategies, such as enzyme replacement therapy, gene therapy, and pharmacological chaperone therapy [18, 19]. One particularly promising approach involves using protective protein/cathepsin A (PPCA), a chaperone that aids in the lysosomal stability and catalytic activation of *NEU1*, can increase *NEU1* activity and improve disease phenotypes in mutant mice with type I sialidosis [20, 21]. Despite the promising results from preclinical studies, the recent success of gene therapy for other inherited diseases offers hope for future treatment options for patients with sialidosis.

## Conclusions

In conclusion, we identified a homozygous *NEU1* mutation (NM_000434.3:c.544 A > G[p.Ser182Gly]) in three siblings diagnosed with type I sialidosis. A heterozygous *NEU1* mutation was also found in their co-parents and the next generation. Thus, with the ongoing advancement of genomic research, rapid and accurate diagnosis of type I sialidosis is becoming increasingly feasible. Furthermore, gene sequencing plays a crucial role in genetic counseling for families affected by type I sialidosis. It also provides a solid foundation for the future research of effective drugs and precision treatments, including gene intervention.

## Data Availability

The datasets used and analyzed during the current study are available from the corresponding author upon reasonable request.

## Abbreviations

AMPAR: α-Amino-3-hydroxy-5-methyl-4-isoxazole-propionate receptor
CSF: Cerebralspinal fluid
MRI: Magnetic resonance imaging
NEU1: Neuraminidase-1
PPCA: Protective protein/cathepsin A
